# Errata

**Published:** 1782

**Authors:** 


					>. 436/A* 13, f.r^msjy. Prof, read Ms D.^rwf,
END of VOL. Ill,

				

